# Spinal cord injury in high-risk complex adult spinal deformity surgery: review of incidence and outcomes from the Scoli-RISK-1 study

**DOI:** 10.1038/s41394-024-00673-y

**Published:** 2024-08-17

**Authors:** Fan Jiang, Hetshree Joshi, Jetan H. Badhiwala, Jamie R. F. Wilson, Lawrence G. Lenke, Christopher I. Shaffrey, Kenneth M. C. Cheung, Leah Y. Carreon, Mark B. Dekutoski, Frank J. Schwab, Oheneba Boachie-Adjei, Khaled M. Kebaish, Christopher P. Ames, Sigurd H. Berven, Yong Qiu, Yukihiro Matsuyama, Benny T. Dahl, Hossein Mehdian, Ferran Pellisé, Stephen J. Lewis, Michael G. Fehlings

**Affiliations:** 1 Department of Surgery and Spine Program, University of Toronto, Toronto, ON Canada; 2 Department of Neurosurgery, University of Nebraska Medical Center, Omaha, NE USA; 3 Department of Orthopedic Surgery, The Spine Hospital, Columbia University Medical Center, New York, NY USA; 4 Department of Orthopaedic Surgery, Duke University, Durham, NC USA; 5 Department of Orthopaedics and Traumatology, The University of Hong Kong, Pokfulam, Hong Kong SAR, China; 6 Norton Leatherman Spine Center, Louisville, KY USA; 7 The CORE Institute, Sun City West, AZ USA; 8 Spine Service, Hospital for Special Surgery, New York, NY USA; 9 The FOCOS Hospital, Pantang West, Republic of Ghana; 10 Department of Orthopaedic Surgery, Johns Hopkins Hospital, Baltimore, MD USA; 11 Department of Neurological Surgery, University of California, San Francisco, San Francisco, CA USA; 12 Department of Orthopaedic Surgery, University of California, San Francisco, San Francisco, CA USA; 13 Spine Surgery, Drum Tower Hospital of Nanjing University Medical School, Nanjing, Jiangsu China; 14 Department of Orthopedic Surgery, Hamamatsu University School of Medicine, Hamamatsu, Shizuoka Japan; 15 Division of Orthopedic Surgery, Texas Children’s Hospital, Baylor College of Medicine, Houston & Rigshospitalet, National University of Denmark, Copenhagen, Denmark; 16 The Centre for Spinal Studies and Surgery, Queen’s Medical Centre, Nottingham University Hospitals, Nottingham, UK; 17 Hospital Universitari de la Vall d’Hebron, Barcelona, Spain

**Keywords:** Outcomes research, Spinal cord diseases

## Abstract

**Study design:**

Clinical case series.

**Objective:**

To describe the cause, treatment and outcome of 6 cases of perioperative spinal cord injury (SCI) in high-risk adult deformity surgery.

**Setting:**

Adult spinal deformity patients were enrolled in the multi-center Scoli-RISK-1 cohort study.

**Methods:**

A total of 272 patients who underwent complex adult deformity surgery were enrolled in the prospective, multi-center Scoli-RISK-1 cohort study. Clinical follow up data were available up to a maximum of 2 years after index surgery. Cases of perioperative SCI were identified and an extensive case review was performed.

**Results:**

Six individuals with SCI were identified from the Scoli-RISK-1 database (2.2%). Two cases occurred intraoperatively and four cases occurred postoperatively. The first case was an incomplete SCI due to a direct intraoperative insult and was treated postoperatively with Riluzole. The second SCI case was caused by a compression injury due to overcorrection of the deformity. Three cases of incomplete SCI occurred; one case of postoperative hematoma, one case of proximal junctional kyphosis (PJK) and one case of adjacent segment disc herniation. All cases of post-operative incomplete SCI were managed with revision decompression and resulted in excellent clinical recovery. One case of incomplete SCI resulted from infection and PJK. The patient’s treatment was complicated by a delay in revision and the patient suffered persistent neurological deficits up to six weeks following the onset of SCI.

**Conclusion:**

Despite the low incidence in high-risk adult deformity surgeries, perioperative SCI can result in devastating consequences. Thus, appropriate postoperative care, follow up and timely management of SCI are essential.

## Introduction

Perioperative spinal cord injury (SCI) is an intrinsic risk to any surgery involving the spine at the spinal cord level. The injury can result from any direct, indirect, or ischemic physiologic insult to the spinal cord intraoperatively or immediately postoperatively, leading to temporary or permanent neuronal dysfunction and impairment [1]. In addition to the devastating physical consequences, the psychological effect of this injury as well as the lifetime costs of care for SCI patients pose a significant societal and healthcare burden [2–6].

Adult spinal deformity (ASD) is a heterogenous family of conditions encompassing a broad spectrum of underlying etiology with varying severity, and can involve deformity in either the coronal, sagittal, or axial plane [7–11]. Although not all ASD patients are symptomatic, in severe cases the spinal imbalance as well as compression of neurological elements can cause significant functional limitation and reduced quality of life [7, 12–14]. Despite advancements in the field of spine surgery, the reported complication rates in adult deformity surgery remain considerably high, reportedly ranging from 10.5% to 96% [15–17]. Previous studies have reported substantial variability in the risk of perioperative neurological complications in spinal deformity surgery, with incidences varying between 0.69% and 5% [18–23]. However, due to limitations in the rigor of neurological assessments, the actual prevalence of perioperative SCI is much less certain with rates reportedly <1% [19–22], although it is recognized that this rate may be under-reported.

To address these knowledge gaps, our group undertook the Scoli-RISK-1 study, a multicenter, prospective cohort study comprised of patients who underwent surgical correction for complex ASD [17, 24–27]. Previous research based on this database has reported lower extremity motor score (LEMS) decline in 23% of patients at discharge following high-risk deformity corrections [28]. However, to date we have not explicitly focused on an analysis of perioperative SCI. Therefore, the objective of this study was to assess the incidence and spectrum of perioperative SCI in complex high-risk ASD surgeries in the Scoli-RISK-1 cohort, as well as to present the identified cases as a case series to further illustrate the etiological causes, treatment options and eventual outcomes.

## Material and methods

### Patient population

Scoli-RISK-1 enrolled surgical patients with complex ASD from September 2011 to September 2012. The inclusion criteria limited the recruitment of patients to the age range between 18 and 80 years old with complex cervicothoracic or thoracolumbar deformity, between C7 and L2. In addition, one or more of the following criteria had to be met to be considered a high-risk procedure: spinal curvature with major Cobb angle in the coronal or sagittal plane of ≥80°; the necessity of using corrective osteotomies for congenital deformity or revision procedures, requirement of three-column osteotomies (pedicle subtraction osteotomy (PSO), vertebral column osteotomy (VCR)); diagnosis of myelopathy in the presence of spinal deformity requiring reconstruction, or deformity correction with concurrent ossification of the ligamentum flavum (OLF) or ossification of the posterior longitudinal ligament (OPLL) causing secondary spinal cord compression. Any patients with a history of substance dependency, psychosocial disturbance, active malignancy, active infection, a recent history of trauma/malignancy in the spine, long-term complete paraplegia, active pregnancy, prisoners and other institutionalized individuals were considered ineligible for this study and were thus excluded.

The Scoli-RISK-1 database included patients recruited from 15 specialized centers, nine from North America, three from Europe and three from Asia. Approval for the study was obtained from each site’s ethics board. Informed consent was obtained before enrollment for each patient. All methods were performed in accordance with the relevant guidelines and regulations. The surgical approach, methods of instrumentation, corrective techniques and use of intraoperative neurological monitoring were at the discretion of the primary treating surgeon.

### Data collection

Preoperatively, patient demographical data was obtained along with preoperative upright x-ray orthogonal images in both anterior-posterior as well as lateral views. Operative data were collected including levels of instrumentation, osteotomy, interbody insertion, blood loss, and relevant intraoperative events. Neurological outcomes were followed primarily using the International Standards for Neurological Classification of Spinal Cord Injury (ISNCSI). The lower extremity motor score (LEMS) has been previously validated for the assessment of ambulatory capacity in patients who sustained incomplete SCI [29]. With the baseline set within six weeks prior to operation, the postoperative exams were performed at the time of discharge, six weeks, six months and two years follow up. Adverse events were reported by the responsible surgeons and documented along with the known or suspected etiology, course of action, and outcomes.

### Retrospective search

An extensive search of the Scoli-RISK-1 database was performed to identify patients with documented perioperative SCI. Our definition of the “perioperative” timeframe begins intraoperatively during the initial surgery and extends up to one year postoperatively. A detailed review of patient data including the type of procedure, the triggering event, the medical or surgical management as well as the final clinical outcome was performed and summarized.

## Results

A total of six cases (2.2%) of perioperative SCI were found in the 272 patients enrolled in the Scoli-RISK-1 study (Table 1).

**Table 1:** Summary of cases with procedures, cause and severity of spinal cord injury as well as treatment and outcomes.

No.	Patient	Procedure(s)	Cause of SCI	Level of SCI	Treatment	AIS at discharge	Outcomes
1	48-year-old male	Single stage: T2-L2 PSF and T7 VCR	Intraoperative direct injury	T9 incomplete	Riluzole	AIS grade D	Recovered to preoperative baseline at 6 months postoperatively
2	57-year-old male	Stage 1: T1-pelvis PSF, with T2–T3 and T3–T4 SPO Stage 2: L3 PSO, L2–L3 interbody insertion, and revision of T11-pelvis instrumentation and PSF	Overcorrection of deformity after Stage 1 procedure	T3 incomplete	Revision and release of correction	AIS grade D	Significant neurological improvement occurred postoperatively following the revision. Stage 2 procedure proceeded as planned without complications. LEMS at discharge was 23 bilaterally in the lower extremities, AIS D. However, the deficit was persistent at six months follow up
3	70-year-old female	Single-stage: T9-pelvis with T12 VCR	Postoperative hematoma	T12 incomplete	Revision decompression and evacuation of the hematoma	AIS grade E	Postoperative complete recovery
4	60-year-old female	Stage 1: T8-pelvis PSF with T12 and L1 VCR Stage 2: Extension of fusion to T3 with T7 PSO and T6–T7 interbody insertion	PJK	T2 incomplete	Revision with correction of alignment and extension of fusion to C4	AIS grade E	Postoperative recovery to baseline neurological function
5	52-year-old male	Single-stage: T9-pelvis PSF with SPO at T11–T12, T12–L1, L1–L2 and L2–L3, VCR at L3 and L4, and Interbody insertion at L5–S1	Disk herniation T8–T9 level	T8 incomplete	Surgical decompression	AIS grade E	Postoperatively full neurological recovery
6	60-year-old male	Single-Stage: T10–S1 PSF with L2 PSO	T9–T10 discitis, osteomyelitis, development of phlegmon and PJK	T10 incomplete	Initial revision decompression with T9 laminectomy, irrigation and washout of the surgical site. Due to persistent compression of the spinal cord on MRI, a second revision was planned but delayed due to medical reasons. An osteotomy at T9–T10 and extension of fusion to T4 was eventually performed two weeks following the onset of SCI	AIS grade C	Persistent motor and sensory impairments in bilateral lower extremities at the six weeks postoperative visit. Further details concerning recovery were not available due to the patient’s withdrawal from the study

*SCI* spinal cord injury, *AIS* ASIA Impairment Scale, *PSF* posterior spinal fusion, *VCR* vertebral column resection, *SPO* Smith-Peterson osteotomy, *PSO* pedicle subtraction osteotomy, *PJK* proximal junctional kyphosis, *MRI* magnetic resonance imaging.

### Case 1

**Fig. 1:**
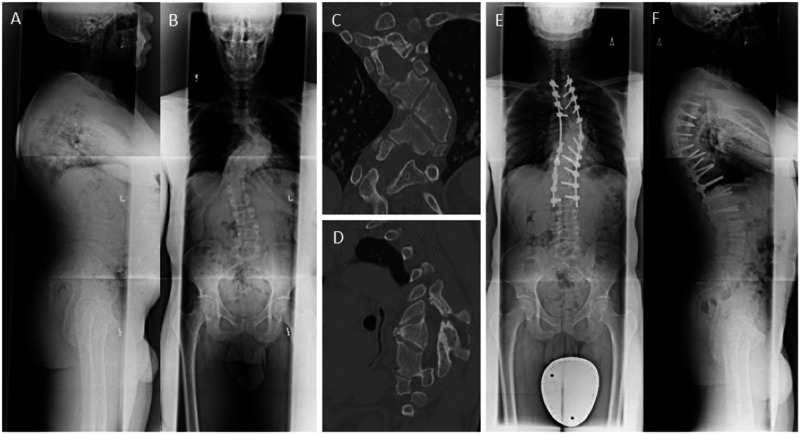
Case 1, a 48-year-old healthy man with a chief complaint of mid-thoracic and low back pain with a history of congenital scoliosis diagnosed at 12 years of age. **A** Anterior-posterior (AP) and (**B**) lateral standing radiographs demonstrated a kyphoscoliotic deformity localized at the main thoracic level, measuring 84° on Cobb angle coronally. Selective computed tomography (**C**) coronal and (**D**) sagittal images revealed unsegmented hemivertebrae at the T7 and T8 levels. **E**, **F** The patient underwent a T7 vertebral column resection (VCR) and T2-L2 posterior spinal fusion. The procedure was complicated by the intraoperative bilateral loss of motor evoked potentials. Postoperatively, the patient woke with complete loss of power in bilateral lower extremities and preservation of sensation. He was diagnosed with a T9 incomplete SCI and treatment with Riluzole was begun immediately. On the day of discharge, his lower limb power improved to 3/5 motor power bilaterally in all muscle groups. He made a significant recovery in rehabilitation and at 6 months follow up he was ambulatory with grade 5/5 motor strength in bilateral lower extremities.

A 48-year-old male with a history of congenital scoliosis and no preoperative neurological deficit underwent a complex T2-L2 posterior spinal fusion with T7 VCR (Fig. 1). Intraoperatively, while working under the spinal cord performing the vertebrectomy, there was a loss of motor evoked potentials bilaterally. After ensuring that there were no compressive elements around the spinal cord, the surgeons went ahead and completed the closure of the vertebrectomy. Postoperative examination revealed 0/5 motor power in bilateral lower limbs with preservation of sensation to pinprick and light touch, in keeping with a T9 incomplete SCI. The patient was treated medically with Riluzole, starting within 12 hours following surgery. For the first 24 hours, 100 mg of Riluzole was given every 12 hours by mouth. Following that Riluzole was continued for 2 weeks at a dosage of 50 mg twice a day. He began experiencing neurological recovery during hospitalization and was discharged to rehabilitation. The patient’s LEMS was 15/50 for the lower limbs bilaterally at the time of discharge. He experienced significant recovery over the ensuing months and his neurological exam improved to his preoperative baseline (50/50) at six months follow up.

### Case 2

A 57-year-old male with a known history of hypertension, osteoarthritis, and previous spinal fusion procedure elected to undergo a staged corrective operation for ASD. The patient initially presented without any neurological deficit. The staged procedure was carried out with stage 1 including T2-T3 and T3-4 Smith-Peterson osteotomy (SPO) with pedicle screw and temporary rod stabilization from T1 to the pelvis. Given the extent of the surgery, intraoperative blood loss was controlled and kept to an appropriate level. Postoperatively, a slowly progressive loss of motor function in the lower extremities began 12 hours after surgery. Examination revealed an incomplete SCI at the T3 neurological level. Upon investigation, the cause of the neurological deficit was determined to be overcorrection of the deformity, thus the decision was then made by the primary treating surgeon to return to the operating room to release the correction to the proximal thoracic spine. The patient was urgently taken back to the OR within the same day and immediate postoperative improvement was noted in neurological function. The patient returned to the operating room two weeks later for the 2nd stage whereby the pedicle screw and hook construct was then completed along with insertion of interbody cage at L2-3 and an L3 PSO for the final correction. The procedure was uncomplicated, and the patient was discharged to rehabilitation with a LEMS of 23 in the lower extremities and normal sensation. At the six-month follow up visit, persistent lower limb motor and sensory deficits were noted.

### Case 3

A 70-year-old female underwent a single-stage T9 to pelvis posterior spinal fusion procedure with T12 VCR and cage reconstruction. The surgical procedure was uncomplicated. However, on postoperative day 1, the patient was found to have weakness bilaterally in the lower extremities associated with decreased sensation. Physical examination revealed 1/5 motor power in hip flexion, knee extension, ankle dorsi and plantar flexion consistent with an incomplete American Spinal Injury Association Impairment Scale (AIS) grade C. Immediate CT scan revealed a compressive epidural hematoma at the T12 level. Revision and evacuation of the hematoma was performed on an urgent basis and the patient made a complete recovery of her motor as well as sensory deficits and was discharged home.

### Case 4

A 60-year-old female with a history of non-metastatic cancer, osteoporosis, hypertension and anxiety along with three previous spinal procedures underwent a staged correction for her ASD. The first stage of the operation included T8 to pelvis posterior spinal fusion with VCR of T12 and L1, as well as reconstruction with cage implant. The second stage followed a period of recovery with an extension of the fusion to T3, a PSO at the level of T7 and insertion of an interbody cage at the T6-T7 level. At nine days postoperatively, she experienced electric shock-like sensations in the legs accompanied by the onset of lower extremity weakness. Investigation revealed an incomplete T2 SCI secondary to proximal junctional kyphosis. Emergent surgical revision was performed for deformity correction, decompression, and extension of fusion to C4. The patient recovered with resolution of symptoms and a return of neurological function to baseline.

### Case 5

A 52-year-old male presented with a chief complaint of bilateral lower extremity weakness and gait imbalance. He was diagnosed with congenital deformity causing myelopathy. Aside from known hypertension, he was otherwise in good health. However, his history was complicated by two previous spinal decompression and fusion procedures. Preoperatively, he had a mild left lower extremity sensory deficit to light touch and intact LEMS on examination.

The patient underwent T9 to pelvis posterior spinal fusion, with 4 level SPO at T11-T12, T12-L1, L1-L2, L2-L3, 2 level VCR at L3 and L4 with cage reconstruction, and L5-S1 interbody cage insertion. The operation was complicated by excessive bleeding and the development of an intraoperative extensive erythematous rash of unknown etiology, likely an allergic reaction. Additionally, postoperatively the patient developed anemia, thrombocytopenia, and a dental abscess. Despite this, the patient was neurologically stable with no change to the slight sensory deficit present before the operation and was discharged to home.

At five months postoperatively, the patient reported numbness in the right leg with difficulty ambulating and increased urinary frequency. An x-ray confirmed a fracture of the rod. However, given his symptoms were improving, a surgical revision was planned for a later date. At nine months post indexed surgery, the patient fell while getting out of bed. Initially the patient was able to move his legs, however, two hours later he woke up with complete loss of motor function in bilateral lower extremities. Upon arrival at the specialized center, he was found to have a T8 incomplete SCI. Steroids were given and the patient was taken for an urgent MRI which revealed a disc herniation above the fusion construct. He was brought to the operating room for surgical decompression. Postoperatively, despite the complication of postoperative osteomyelitis, neurological status improved, and motor strength improved to 5/5 in assessed muscles of the lower extremities.

### Case 6

A 60-year-old male with a medical history of degenerative arthritis complicated by multiple previous spinal operations, underwent a T10 to S1 posterior spinal fusion and L2 PSO. Surgery was uncomplicated and the patient was discharged to home. At five weeks after surgery, he presented with neurological deficits consistent with a T10 incomplete SCI secondary to T9-T10 discitis and epidural abscess. The patient underwent an urgent revision T9 laminectomy with irrigation and debridement of the infection. Postoperatively, due to persistent neurological deficit, a repeat MRI scan was performed revealing a collapse of the T9-T10 disc space causing PJK, as well as the demonstration of an intraosseous abscess and epidural phlegmon resulting in significant compression to the spinal cord. Unfortunately, surgical revision was delayed due to medical reasons. At two weeks following the previous revision procedure, the patient was taken to the operating room for a second repeat decompression with an osteotomy at the level of T9-T10 and extension of the fusion to T4. The patient was discharged to rehabilitation with a persistent neurological deficit. At the six weeks postoperative visit, his motor and sensory loss remained unchanged, with a lower extremity motor score of 22/50 and total sensory scores of 64/100 for both light touch and pin prick. Unfortunately, the patient withdrew consent and no further information on his recovery was available.

## Discussion

This study reflects the high risk of complex deformity procedures and provides examples of common causes of perioperative SCI. To address the knowledge gap in this area, this retrospective review provided concrete examples of how perioperative SCI was dealt with by spinal deformity experts.

In this study, using the international, multicentered Scoli-RISK-1 database, we focused on the group of ASD patients with complex spinal deformities. The rate of perioperative SCI in this cohort was found to be 2.2%, comparatively higher than rates which are reported in the literature for typical deformity surgery [19–22], but not exceeding the reported rate for SCI in advanced posterior column osteotomies [16, 30–32]. Although the overall risk for SCI in our cohort is seemingly low, it is essential to note that the patients enrolled in the Scoli-RISK-1 study were treated by spine surgeons with both expertise and experience in spinal deformity procedures, and who worked at specialized institutions. Therefore, the results of this study should not be generalized to all deformity procedures and contexts, and therefore need to be interpreted with caution. Furthermore, we strongly suggest that high-risk complex deformity procedures be performed by surgeons with the proper training and expertise at institutions with adequate resources and experience.

In this series, intraoperative events accounted for one case of SCI, due to direct trauma to the spinal cord. Postoperative adverse events were responsible for the other five, and were comprised of progressive neurological deficit due to overcorrection of deformity, a postoperative compressive hematoma, an adjacent segment disk herniation, a PJK, as well as one case of discitis leading to osteomyelitis, abscess, epidural phlegmon and PJK. Of these six cases, only one case was managed medically, while the others were revised surgically. Five of the six patients demonstrated significant recovery with the prompt treatment of their SCI. In one patient, a delay in surgical revision occurred due to medical reasons. Despite adequate decompression, his motor and sensory deficits remained at the six weeks follow up visit. However, the patient withdrew from the study and no further details concerning his recovery were available. Therefore, this clinical series illustrates the need for clinical vigilance in the perioperative period following complex deformity correction. Additionally, in keeping with the existing literature, this study further demonstrated the urgency for reversal of causative intraoperative maneuvers or revision and decompression of postoperatively identified compressive lesions when appropriate, as determined by the patient’s medical status [1, 19, 22].

As noted in this case series, the administration of corticosteroids in the event of SCI was left to the clinical judgment of the treating surgeon. Due to the previously reported increased risk of infection, the use of methylprednisolone in this context remains inconsistent in the spine community [33–35]. However, recent evidence-based guidelines supported by a robust systematic review of the literature demonstrated the benefit of using corticosteroids when administered within 8 hours of acute traumatic SCI with no significant difference in the rate of complications [36, 37].

In the event where no revisable spinal cord compression is identified, the option of medical treatment and observation has been reported [1, 22]. Aside from corticosteroids, a few other agents have been tried and are in various stages of testing or clinical trials [35, 38, 39]. The medically managed case presented here was treated with Riluzole, a voltage-gated sodium channel blocker used previously in the treatment of amyotrophic lateral sclerosis [40]. Riluzole has shown promise in providing neuroprotection and promotes functional recovery after SCI in animal models [41, 42]. Although the application or Riluzole for neuroprotective purposes in SCI is relatively new, early clinical trials in humans are showing promising results [43, 44].

The multicentered randomized controlled trial, Riluzole in Acute Spinal Cord Injury Study (RISCIS), commenced in 2014 [45] with the primary outcome targeted at assessing change in upper and lower extremity motor scores. Due to the global COVID pandemic, the Phase III RISCIS trial was stopped in 2021 prior to reaching the pre-planned patient enrollment [46]. Regardless, with the 193 subjects (54.9% enrollment) randomized to treatment and placebo arm, Fehlings et al., 2023 [46], reported improvement in the gain of 1.76 upper extremity motor and 2.86 total motor score in the Riluzole-treated subjects at 180 days. However, the results did not reach statistical significance. While the primary analysis of the RISCIS trial did not achieve predetermined endpoint efficacy, likely related to insufficient power, secondary analysis of other measures, including SCIM, SF36, EQ5D, GRASSP and neurological levels gained, also showed no statistically significant improvement in the treatment group. However, in the AIS B subgroup, Riluzole-treated patients experienced statistically significant improvements in the SF36 mental component and the SCIM total score, respiratory management and self-care component. Despite these new findings, the usage of Riluzole in the context of SCI is still new and requires further investigation. Although we presented the case of SCI treated non-surgically, we cannot attribute the neurological recovery to Riluzole administration. Hence, we are not advocating for the use of Riluzole in SCI but simply presenting this as an alternative option in cases where surgical intervention may not be possible or indicated.

Finally, there is still a lack of consensus in the prevention and management of intraoperative SCI during spinal surgeries. Recognizing this critical knowledge gap, recently, a focused group of international SCI experts systematically reviewed the current literature and formulated the evidence into clinical practice guidelines [47–49]. Using the GRADE protocol, the group recommends prompt identification of patients undergoing surgical procedures at high risk of intraoperative SCI, early involvement of multi-disciplinary care team, and use of intraoperative neuromonitoring during the operation [49]. The AO Spine PRAXIS care pathway was concurrently developed using an evidence-based approach to serve as a reference for the perioperative management of high-risk spine surgery patients. The guideline also includes an extensive checklist, modified from Vitale et al. 2014, to guide and assist surgeons in the event of intraoperative neuromonitoring signal changes [48, 50]. With these novel recommendations and guidelines, we anticipate future prospective multicentered studies to validate and assess their efficacy in reducing intraoperative SCI.

### Study limitations

This study is subject to several limitations. Although the strict inclusion criteria allowed for the enrollment of patients with complex ASD, it does not select for a specific underlying diagnosis. Hence, the study cohort is still heterogeneous in terms of the etiology of deformities. Similarly, the surgical procedures for spinal deformities as well as the treatment protocol for perioperative SCI were not standardized, further highlighting the need for evidence-based guidelines to manage perioperative SCI in ASD surgery. Additionally, since the Scoli-RISK-1 cohort included only adult patients who underwent high-risk spinal deformity procedures, the incidence of perioperative SCI reported in this case series needs to be interpreted with caution when being applied to the general spinal deformity patient population. Furthermore, given the rarity of perioperative SCI, the small number of patients precluded the use of statistical analysis to assess for predictors and risk factors. Finally, given the nature of the database, our description of the cases is limited to the available data. Therefore, certain details including exact neurological deficit at the onset of adverse events, the exact nature of the injury and diagnostic imaging performed are not available. However, a future detailed chart review of each case would be of benefit to further dissect and understand each event.

## Conclusion

Although the complication rate specific to perioperative SCI remains low in complex high-risk deformity surgeries, this case series highlights the variability in the etiology, operative management, and recovery with medical or surgical treatment. Despite the relatively low incidence, perioperative SCI can result in possible permanent neurological deficits. Thus, it is essential for these procedures to be performed by experienced spine surgeons with skills and expertise in deformity correction. Furthermore, the recovery of these patients varies greatly case by case. We therefore strongly advocate for careful postoperative observation and timely management of SCI to optimize the chance of recovery. Further research, not only with regards to risk factors, but with regards to the course of recovery in these cases is warranted. Finally, this study highlighted the need for standardized clinical practice guidelines for the management of perioperative SCI following complex deformity procedures.

## Data Availability

The data that supports the findings of this study are available from the corresponding author, MF, upon reasonable request.
